# Multilevel Genetic and Functional Assessment of an ARR3 Frameshift Variant in Early‐Onset High Myopia

**DOI:** 10.1155/ijog/8894003

**Published:** 2026-06-20

**Authors:** Yadi Li, Fengwu Teng, Qin Zhu, Shulan Cui, Wenfu Yang, Pengcheng Ma

**Affiliations:** ^1^ Department of Pediatric Ophthalmology, Affiliated Hospital of Yunnan University, Kunming, Yunnan, China, ypfph.com; ^2^ Department of Radiology, Yan′an Hospital Affiliated to Kunming Medical University, Kunming, Yunnan, China; ^3^ Department of Radiology, Wenshan Prefecture People′s Hospital, Wenshan, Yunnan, China

**Keywords:** ARR3 variant, cone arrestin, early-onset high myopia, frameshift mutation, genetic pathogenesis, retinal dysfunction, X-linked myopia

## Abstract

**Background:**

Early‐onset high myopia (eoHM) is a highly heritable ocular disorder, with pathogenic variants in the X‐linked ARR3 gene (which encodes cone arrestin) being associated with a female‐limited form of eoHM. However, the clinical manifestations of early truncating variants in ARR3 and their potential impact on the classic sex‐limited pattern remain insufficiently understood.

**Methods:**

This study examined individuals with eoHM from a multigenerational Chinese family, utilizing whole‐exome sequencing, Sanger validation, and segregation analysis to identify candidate variants. In silico predictions and protein structure modeling were conducted to assess the variant′s pathogenicity. Furthermore, in cultured cells, quantitative real‐time PCR, Western blotting, and fluorescence microscopy were used to evaluate ARR3 transcript expression and ARR3‐related immunodetected signal after transfection with wild‐type or mutant ARR3 constructs, thereby providing a preliminary functional assessment of the variant.

**Results:**

We discovered a novel frameshift variant, c.721delT (p.Y241Ifs∗3), which cosegregated with eoHM in the family and was absent in public population databases. Affected individuals, including hemizygous males, exhibited early‐onset, high‐degree myopia and fundus changes consistent with pathologic myopia. Functional assays in ARPE‐19 cells showed that the mutant construct was associated with markedly reduced ARR3 mRNA expression and decreased ARR3 immunoreactivity compared with the wild‐type construct. Because no direct cellular injury assay was performed, these in vitro findings were interpreted as preliminary expression‐level evidence supporting a likely loss‐of‐function mechanism.

**Conclusion:**

The novel ARR3 frameshift variant c.721delT (p.Y241Ifs∗3) is likely pathogenic for eoHM and may alter the previously understood female‐limited inheritance pattern. These findings expand the known mutation spectrum of ARR3 and enhance understanding of the role of cone arrestin dysfunction in the development of eoHM.

## 1. Introduction

Myopia has become one of the most prevalent refractive errors worldwide, with its prevalence steadily increasing over recent decades, posing a significant public health challenge. Epidemiological projections estimate that by 2050, nearly half of the global population may be affected by myopia, with around 10% developing high myopia. Recent international consensus highlights that myopia, especially high myopia, is a priority global eye health issue, necessitating systematic public health strategies and interdisciplinary collaboration to mitigate its long‐term disease burden [1–3]. High myopia substantially raises the risk of serious ocular complications such as retinal detachment, myopic macular degeneration, choroidal neovascularization, and glaucoma, thereby imposing significant negative impacts on patients′ quality of life, healthcare costs, and workforce productivity [4, 5]. Current interventions, including optical correction, low‐concentration atropine eye drops, and environmental modifications, are primarily aimed at slowing myopia progression but have limited impact on underlying mechanisms such as excessive axial elongation and structural remodeling of the fundus. This has refocused research efforts on the genetic basis and molecular pathology of high myopia to discover more effective prevention and management strategies.

Among high myopia patients, early‐onset high myopia (eoHM) often manifests more distinct genetic predispositions and severe clinical consequences. This form of eoHM typically progresses rapidly during preschool or early elementary school years, with marked axial elongation and ocular structural changes, presenting risks of blindness and disease burden far exceeding those of typical adolescent‐onset myopia [5]. The advent of whole‐exome sequencing and targeted gene panels has led to the identification of numerous pathogenic genes associated with eoHM, including X‐linked and autosomal genes like OPN1LW/OPN1MW, RP2, and GPR143, some of which are also implicated in other hereditary retinal diseases [6, 7]. Of particular interest, ARR3 (cone arrestin) has emerged due to its association with a rare “X‐linked, female‐limited” early‐onset high myopia (MYP26), garnering considerable interest from researchers [8–10]. ARR3 encodes cone arrestin, a visual arrestin enriched in cone photoreceptors that contributes to the timely termination of cone phototransduction and the maintenance of cone function. Structurally, ARR3 contains conserved arrestin domains; disruption of these regions, particularly truncations affecting the downstream C‐terminal region, may compromise protein stability and function. Studies have shown that ARR3‐related eoHM typically features childhood onset, poor response to conventional corrective treatments, high myopia, and manifestations such as tessellated fundus and temporal peripapillary crescent atrophy, with some patients experiencing mild cone dysfunction [11]. Notably, its inheritance pattern is distinct: heterozygous females carrying pathogenic ARR3 variants generally develop eoHM, whereas hemizygous males often exhibit normal refraction or mild myopia and are deemed asymptomatic carriers, which contrasts sharply with the “male‐severe” pattern in classic X‐linked recessive diseases [12]. To explain this “female‐limited” phenomenon, hypotheses suggest that X‐chromosome inactivation in females results in the presence of both wild‐type and mutant ARR3 proteins, causing functional interference; whereas in males, the expression of a single allele results in insufficient function below the pathogenic threshold [13]. However, the mutation and phenotype spectra of ARR3‐related eoHM remain incompletely defined, and there is insufficient evidence to suggest that different variant types may breach the traditional “female‐limited” inheritance mode.

With the broad application of next‐generation sequencing (NGS) and functional genomics technologies, research on monogenic high myopia has progressed beyond mere gene screening to exploring specific pathogenic mechanisms [14]. In the ARR3 gene, several missense, nonsense, and splice‐site variants have been reported, with some studies preliminarily linking phenotypes to genotypes through electrophysiological studies and retinal imaging [15, 16]. However, systematic experimental data are still lacking to clarify how ARR3 variants disrupt key structural domains of cone arrestin, its intracellular localization, and signal termination functions; moreover, a complete mechanistic chain linking these molecular changes to cone cell microenvironment dysregulation, scleral remodeling imbalance, and abnormal axial regulation has yet to be constructed. Specifically concerning frameshift truncation variants in early exons, whether they violate the traditional phenotype pattern of “female‐limited, nonpenetrant in hemizygous males” through severe loss‐of‐function effects remains unverified; additionally, recent findings associating another X‐linked gene, GLRA2, with eoHM suggest that the genetic landscape of such pedigrees may be more complex than initially anticipated [17].

We identified a novel frameshift variant in the ARR3 gene‐c.721delT (p.Y241Ifs∗3)‐within a multigenerational eoHM pedigree from Yunnan, China. In this pedigree, several female members developed high myopia in childhood, showing typical high myopia fundus changes; notably, one hemizygous male patient exhibited refractive errors and posterior pole structural abnormalities comparable with those in female patients. This phenotypic combination suggests that the variant may possess stronger pathogenicity than previously reported ARR3 variants, potentially disrupting the traditional “female‐limited” inheritance mode. Preliminary structural predictions indicate that c.721delT causes a frameshift beginning at the 241st amino acid in the ARR3 protein, resulting in premature translation termination and complete loss of the C‐terminal conserved domain, theoretically constituting a severe truncating loss‐of‐function variant.

We propose the hypothesis that ARR3 c.721delT (p.Y241Ifs∗3) disrupts the structural integrity of cone arrestin and reduces ARR3 expression, thereby contributing to eoHM through a likely loss‐of‐function mechanism that may be sufficient to produce disease manifestations even in hemizygous males. To validate this hypothesis, we conducted analyses from clinical, genetic, and cellular perspectives. Comprehensive ophthalmic examinations were performed on core pedigree members to document the clinical features of ARR3‐related eoHM in this family. Whole‐exome sequencing, paired with Sanger sequencing for segregation analysis, and various bioinformatics tools and protein modeling were employed to assess the pathogenicity and structural impacts of c.721delT (p.Y241Ifs∗3). Additionally, wild‐type and mutant ARR3 expression vectors were constructed and introduced into a human retinal pigment epithelial cell line to assess their effects on ARR3 mRNA expression and ARR3 immunoreactivity, thereby exploring the cellular‐level functional consequences of the variant.

This study combines in vivo (pedigree clinical and genetic analysis) and in vitro (structural prediction and cellular functional experiments) evidence to systematically examine the association between ARR3 c.721delT (p.Y241Ifs∗3) and eoHM, with an emphasis on challenging the traditional “X‐linked female‐limited” inheritance mode and exploring potential loss‐of‐function mechanisms. By conducting an in‐depth analysis of this novel frameshift variant, we aim to enrich the mutation and phenotype spectra of ARR3‐related eoHM, provide additional evidence for the genetic diagnosis and counseling of high myopia, and lay theoretical foundations for future precision interventions targeting ARR3 and its related pathways.

## 2. Method

### 2.1. Recruitment of the High‐Myopia Pedigree and Phenotypic Assessment

This study was approved by the Ethics Committee of the Affiliated Hospital of Yunnan University, and all procedures adhered to relevant ethical guidelines. Family members were recruited following standardized protocols. Each participant underwent a general physical examination to exclude systemic diseases unrelated to ocular conditions, and written informed consent was obtained from all individuals or their legal guardians. Clinical information, including age at onset and family history, was recorded, and a pedigree chart was constructed.

All affected individuals underwent comprehensive ophthalmic evaluation, including: (1) measurement of best‐corrected visual acuity and intraocular pressure and refraction; (2) slit‐lamp examination of the anterior segment and fundus; and (3) fundus photography, macular and optic disc optical coherence tomography (OCT), and B‐scan ultrasonography. Peripheral venous blood samples (8 mL) were collected from affected individuals and their relatives and stored at −80°C until further analysis.

### 2.2. Whole‐Exome Sequencing of Peripheral Blood Samples

Peripheral blood samples were collected from selected family members, and genomic DNA was extracted from leukocytes using standard procedures. The genomic DNA was fragmented to an average size of 180 bp using a Bioruptor sonicator (Diagenode). Paired‐end sequencing libraries were prepared using the DNA Sample Prep Reagent Set 1 (NEBNext) according to Illumina protocols. The DNA libraries were hybridized with GenCap gene panel probes following the manufacturer′s instructions (MyGenostics, Beijing, China), followed by capture, elution, polymerase chain reaction (PCR) amplification, and purification of the hybridized products. Whole‐exome sequencing was performed on an Illumina NovaSeq 6000 platform (Illumina, United States), covering the exonic regions and flanking intronic sequences of the human exome. Sequencing reads were aligned to the human reference genome hg19, where the coverage of target regions and overall sequencing quality were systematically evaluated. During variant calling, copy number variations (CNVs) involving larger segments (two or more consecutive exons) in candidate genes were assessed and confirmed using orthogonal methods such as multiplex ligation‐dependent probe amplification. Variants with a minor allele frequency > 0.001 in public databases, including ESP6500, the 1000 Genomes Project, ExAC, and gnomAD, were filtered out as common polymorphisms. Potential splice site effects were predicted using Human Splicing Finder (HSF) and dbscSNV Version 1.0, whereas the pathogenicity of candidate single‐nucleotide variants was evaluated with in silico prediction tools. Upon sequencing and bioinformatics processing completion, genetic analyses were performed to identify putative disease‐causing variants in this family.

### 2.3. Sanger Sequencing

Targeted regions were amplified by PCR using specific primers, and the PCR products were subjected to bidirectional Sanger sequencing on a DNA sequencer. The resulting sequences were analyzed with SeqMan software to align the reads to the reference sequence and determine the presence or absence of nucleotide substitutions at the sites of interest.

### 2.4. In Silico Pathogenicity Prediction and Protein Structural Analysis

The potential deleteriousness of the variant was assessed using SIFT, PROVEAN (http://provean.jcvi.org/genome_submit_2.php), and MutationTaster (http://www.mutationtaster.org) and PM2 (http://pm2.keymetrics.io), in combination with the American College of Medical Genetics and Genomics (ACMG) guidelines for variant classification [18, 19]. The PM2 criterion was considered during ACMG‐based interpretation because the variant was absent or extremely rare in public population databases. Protein structural modeling and visualization were performed using UCSF Chimera.

### 2.5. Construction of ARR3 Wild‐Type and Mutant Plasmids

Full‐length ARR3 and mutant ARR3 c.721delT (p.Y241Ifs∗3) expression plasmids were synthesized by a commercial provider (Shanghai, China). The plasmid sizes were 1178 and 1166 bp, respectively. Both constructs contained an ampicillin resistance cassette and were tagged with EGFP and Flag. The backbone vector was pCDNA3.1‐CMV‐MCS‐3xFLAG‐hGHpolyA‐EF1a‐EGFP.

### 2.6. Plasmid Extraction

Glycerol stocks containing wild‐type ARR3 and mutant ARR3 c.721delT (p.Y241Ifs∗3) plasmids were streaked onto LB agar plates supplemented with appropriate antibiotics and incubated at 37°C for 12–16 h until single colonies appeared. Positive clones were selected and inoculated into 200 mL of LB broth for overnight culture, followed by centrifugation to pellet the bacteria and removal of the supernatant. Plasmid DNA was extracted from the bacterial pellet using a commercial plasmid extraction kit (DP430, Tiangen Biotech, Shanghai, China) as per the manufacturer′s instructions. A 20‐*μ*L aliquot was submitted for Sanger sequencing to confirm the presence of the desired mutation, while the remaining plasmid DNA was stored at −20°C until further use.

### 2.7. Cell Culture

Human retinal pigment epithelial cells (ARPE‐19; CL‐0026, Sainuo Biological Technology, Wuhan, China) served as the in vitro model. Cells were cultured under standard conditions in complete growth medium consisting of high‐glucose DMEM supplemented with 10% fetal bovine serum and 1% penicillin–streptomycin and maintained in T75 culture flasks at 37°C in a humidified incubator containing 5% CO_2_. Cells at appropriate confluence were passaged at regular intervals. Cell morphology and growth status were monitored microscopically and recorded throughout the experiments. The microscopes used in this study included an inverted phase‐contrast microscope (XDS‐800C, Carl Zeiss, Germany), an inverted fluorescence microscope (Zeiss Axio Vert. A1, Carl Zeiss, Germany), and a laser confocal microscope (LSM 700, Carl Zeiss, Germany).

### 2.8. Cell Transfection

ARPE‐19 cells were seeded into six‐well plates and cultured overnight to reach approximately 70%–80% confluence at the time of transfection. Cells were divided into three groups: wild‐type ARR3 (WT), mutant ARR3 c.721delT (MUT), and negative control (NC). Transfections were performed using Lipofectamine 3000 Transfection Reagent (L3000015, Invitrogen, United States) following the manufacturer′s protocol. After 24 h, transfection efficiency was evaluated with an inverted fluorescence microscope; cells showing green fluorescence were considered successfully transfected, with efficiency in each well exceeding 90%, ensuring suitability for downstream assays.

### 2.9. Real‐Time Quantitative PCR (RT–qPCR)

For RT‐qPCR analysis, cells were transfected 1 day before RNA extraction. Once transfection efficiency met predefined criteria, total RNA was extracted using TRIzol reagent (15596018, Invitrogen, United States). RNA purification was conducted as per the manufacturer′s instructions, and RNA concentration and purity were determined spectrophotometrically. Complementary DNA (cDNA) was synthesized from total RNA using a 5× PrimeScript RT Master Mix kit (Z3100, Promega, United States) with the cycling conditions: 37°C for 15 min, 85°C for 5 s, then held at 4°C. Primer sequences for amplification are listed in Table 1. The resultant cDNA was diluted fivefold and subjected to real‐time PCR using the 2× GoTaq qPCR Master Mix kit (A6001, Promega, United States). The reactions were run on a real‐time PCR system with initial denaturation at 95°C for 30 s, followed by 40 cycles of 95°C for 5 s and 60°C for 34 s. Fluorescence signals were collected at the end of each cycle to obtain Ct values. Relative gene expression levels were calculated using the 2^−*ΔΔ*Ct^ method, and data were employed for subsequent quantitative analysis [20].

**Table 1:** Primer sequences used in this study.

Target gene (primer)	Primer sequence (3 ^′^–5 ^′^)
ARR3 (human, cell line)	Forward primer: AGTCCACTGGCGTCTTCAC Reverse primer: GAGGCATTGCTGATGATCTTGA
GAPDH (human, cell line)	Forward primer: AGAGACTATGTGCGGCTGGTTGT Reverse primer: AGAAGGAAGCGGCGGATGGT

### 2.10. Western Blot Analysis

For Western blotting, cells were transfected 1 day before protein extraction. Once adequate transfection efficiency was confirmed, cells were washed and lysed on ice in 100‐*μ*L lysis buffer containing RIPA buffer (P0013B, Beyotime, Shanghai, China) supplemented with protease inhibitor (P1005, Beyotime) and phosphatase inhibitor (P1097, Beyotime) for 30 min. Lysates were clarified by centrifugation at 14,000 g for 15 min at 4°C. Total protein concentrations were determined using a BCA Protein Assay Kit (P1005, Beyotime). Equal amounts of protein (80 *μ*L) were mixed with 20 *μ*L 5× loading buffer (P0015, Beyotime), boiled for 5 min, and separated by 10% SDS‐PAGE (1010416, Bio‐Rad, United States). Proteins were then transferred onto PVDF membranes (IPVH00010, Merck Millipore, United States). Membranes were blocked in a rapid blocking buffer (P0252, Beyotime) on a rocking platform at room temperature for 10 min and incubated overnight at 4°C with primary antibodies against ARR3 (PA5‐75301, 1:1000, Invitrogen, United States) and GAPDH (MA5‐15738, 1:5000, Invitrogen, United States). Following washing, membranes were incubated with HRP‐conjugated goat antimouse IgG (H+L) secondary antibody (SA00001‐2, 1:5000, Proteintech, China) at room temperature for 1 h. Protein bands were visualized using a high‐sensitivity ECL chemiluminescence kit (PK10002, Proteintech, China) and imaged with a Bio‐Rad gel documentation system (1708195, Bio‐Rad, United States). Band intensities were quantified using ImageJ (Version 1.8.0.345). The relative ARR3 immunoreactive signal was calculated by normalizing the grayscale intensity of the ARR3‐immunoreactive band to the corresponding GAPDH band intensity.

### 2.11. Statistical Analysis

All experimental data were analyzed using SPSS 26.0 (IBM Corp., Armonk, New York, United States), ImageJ and GraphPad Prism 9 (GraphPad Software, San Diego, California, United States). Each experiment was performed at least three times independently. Differences between groups were evaluated by one‐way analysis of variance (ANOVA). Statistical significance was denoted as follows: ns, *p* > 0.05;  ^∗^ *p* < 0.05;  ^∗∗^ *p* < 0.01;  ^∗∗∗^ *p* < 0.001;  ^∗∗∗∗^ *p* < 0.0001.

## 3. Result

### 3.1. Clinical Identification of an ARR3 Frameshift Variant in a Family With High Myopia

**Figure 1:**
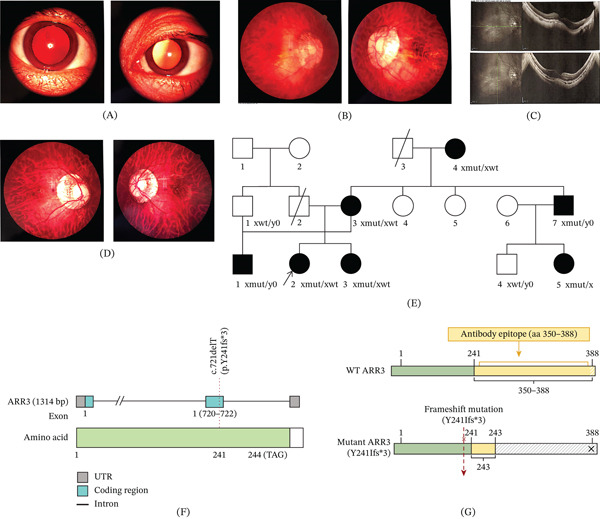
Clinical and genetic findings associated with the ARR3 variant in a family with high myopia. (A) Anterior segment photograph of the proband showing a vacuole‐like change of the crystalline lens in the right eye. (B) Fundus photographs of the proband demonstrating leopard‐spot fundus changes in both eyes (white arrows) and an abnormal foveal reflex in the left macular region. (C) Macular OCT of the proband′s left eye revealing foveal retinoschisis and disorganization of the photoreceptor layer (white arrows). (D) Fundus photographs of an affected family member showing bilateral leopard‐spot fundus changes and a typical myopic choroidal atrophic crescent in high myopia (white arrows). (E) Pedigree and genotype distribution of the family. Genotypes were annotated according to the recommended nomenclature for X‐linked genes. In this notation, xwt/y0 and xmut/y0 denote wild‐type and mutant hemizygous males, respectively, whereas xwt/xwt and xmut/xwt denote wild‐type and heterozygous mutant females, respectively. (F) Schematic representation of the ARR3 gene structure, the encoded cone arrestin protein domains and the location of the c.721delT (p.Y241Ifs∗3) variant. (G) Schematic diagram showing the wild‐type and predicted truncated ARR3 proteins, including the variant position, the affected downstream C‐terminal region, and the antibody‐recognition epitope located at amino acids 350–388.

To investigate the relationship between high myopia and potential genetic variants, we analyzed this family at the levels of clinical phenotype, inheritance pattern, and molecular findings. We identified a multigeneration family with high myopia, in which eight key members underwent detailed ophthalmic examinations and peripheral blood sampling (clinical data in Table 2). Clinically, the proband′s right eye showed vacuole‐like changes of the crystalline lens (Figure 1A). Fundus photography revealed a tigroid fundus pattern in both eyes with patchy chorioretinal changes (white arrows) and an abnormal foveal reflex in the left eye (Figure 1B). Further imaging confirmed posterior segment abnormalities. Macular OCT of the proband′s left eye showed foveoschisis with photoreceptor layer disorganization (Figure 1C), whereas fundus photography of an affected family member revealed bilateral leopard‐spot fundus changes and a typical myopic crescent of choroidal atrophy (Figure 1D).

**Table 2:** Clinical characteristics and mutation status of family members.

Member	Sex	Age	Age at onset	Genotype	Refractive error	Best‐corrected visual acuity	Axial length (mm)	Anterior/posterior segment findings
*Ι*‐4	F	67	Birth	xmut/xwt	R:NA; L:NA	R:0.02; L:0.25	R:29.38; L:29.62	Marked lens opacity (+++); fundus not clearly visualized
II‐1	M	40	—	xwt/y0	NA	R:1.2; L:1.2	NA	—
II‐3	F	37	Birth	xmut/xwt	R:−22.50DS; L:−20.00DS	R:0.25; L:0.25	R:31.26; L:30.88	Lens vacuole‐like changes and foveoschisis with disorganization of the photoreceptor layer
II‐7	M	35	19ages	xmut/y0	R:−7.25DS; L:−9.25DS	R:0.3; L:0.4	R:27.75; L:28.93	Leopard‐spot fundus
III‐1	M	4	—	xmut/y0	R:−2.50DS; L:−1.00DS	R:0.5; L:0.6	NA	Vitreous opacity
III‐2	F	20+	Birth	xmut/xwt	Severe myopia reported by telephone follow‐up, approximately −15.00 DS	NA	NA	NA
III‐3	F	9	Birth	xmut/xwt	R:−15.50DS; L:−16.00DS	R:0.3; L:0.15	R:29.58; L:29.68	Lens and vitreous opacities
III‐4	M	10	—	xwt/y0	R:−0.75DS; L:−0.75DS	R:0.8; L:0.9	R:23.29; L:23.46	—
III‐5	F	2	Birth	xmut/xwt	NA	NA	NA	Direct fundus examination: leopard‐spot fundus

Abbreviation: NA, not available.

Based on the clinical information, we reconstructed the family pedigree (Figure 1E). Affected individuals were observed in multiple successive generations, and both males and females were involved, indicating a dominant pattern of inheritance that, at first glance, resembles autosomal dominant transmission. At the molecular level, we identified a frameshift variant, c.721delT (p.Y241Ifs∗3), in the ARR3 gene (Figure 1F). This variant introduces a frameshift and a premature stop codon, resulting in a truncated protein and is presumed to represent the molecular basis of disease in this family. Taken together, these clinical and genetic findings support a likely association between high myopia and the ARR3 c.721delT (p.Y241Ifs∗3) variant, with this variant plausibly contributing to characteristic structural and functional damage of the eye that drives the development of high myopia.

### 3.2. Conformational Disruption of ARR3 by a Frameshift Mutation and Its Contribution to Disease Progression

To further characterize the distribution of the ARR3 variant within the family, we performed Sanger sequencing. The c.721delT (p.Y241Ifs∗3) variant was shown to cosegregate with the disease phenotype in all eight examined family members (Figure 2A–C), indicating the presence of an ARR3 c.721delT mutation in this pedigree. Alignment of mutant and wild‐type sequences revealed a 1‐bp deletion in Exon 5 of ARR3, corresponding to the NM_004312.3 transcript. This deletion is predicted to replace the original tyrosine at Codon 241 (Y241) with isoleucine (I) and introduce a frameshift that generates a premature termination codon shortly downstream (Figure 2D–E). Thus, the variant not only changes a single amino acid but also truncates the protein by prematurely terminating translation.

Figure 2Structural impact of the ARR3 c.721delT (p.Y241Ifs∗3) frameshift mutation. (A) Sanger sequencing chromatogram confirming the heterozygous ARR3 c.721delT (p.Y241Ifs∗3) variant. (B) Sanger sequencing chromatogram showing the hemizygous ARR3 c.721delT (p.Y241Ifs∗3) variant. (C) Wild‐type Sanger sequencing chromatogram without the c.721delT variant. (D–E) Schematic representation of the nucleotide and protein changes caused by the c.721delT (p.Y241Ifs∗3) mutation, illustrating the 1‐bp deletion, frameshift and introduction of a premature stop codon. (F) Predicted three‐dimensional structure of the wild‐type ARR3 protein, in which Residues 1–241 are rendered in green and Residues 241–288 in orange. (G) Predicted structure of the mutant ARR3 protein after the c.721delT (p.Y241Ifs∗3) mutation, showing loss of Residues 241–288 and truncation of the C‐terminal region. (H) Multiple sequence alignment of ARR3 across different species showing that the tyrosine at Position 241 (Y241) lies within a highly conserved domain.

**Figure:**
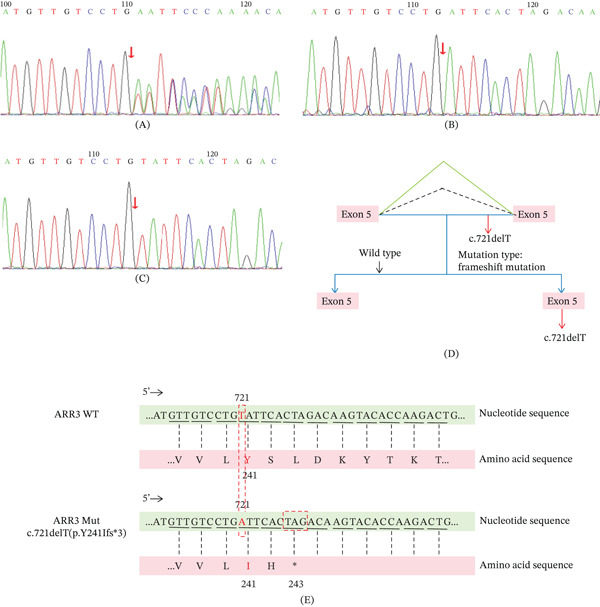
(a)

**Figure:**
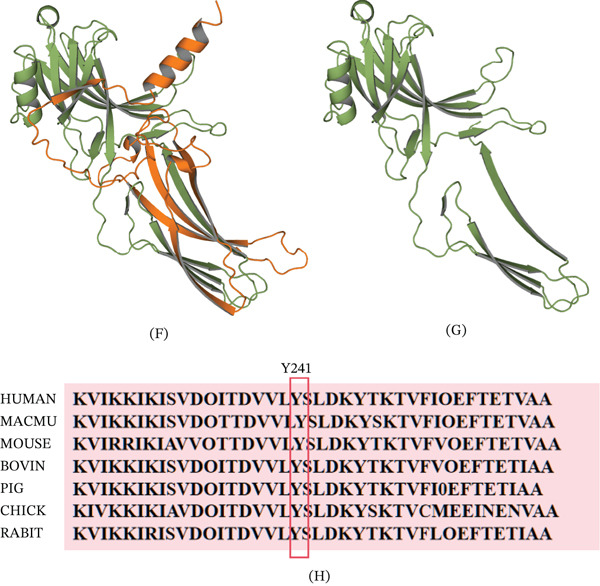
(b)

The c.721delT (p.Y241Ifs∗3) site represents a frameshift mutation in ARR3. To date, five myopia‐associated ARR3 variants have been reported, but none have been frameshift mutations [21]. The c.721delT variant is absent from public databases such as ClinVar and the 1000 Genomes Project. In silico pathogenicity prediction using SIFT, PROVEAN, MutationTaster and PM2, combined with the ACMG variant classification guidelines, indicated that this is a rare and deleterious variant in the general population (Table 3).

**Table 3:** Predicted pathogenicity of the ARR3 c.721delT (p.Y241Ifs∗3) variant.

Mutation site	MutationTaster	PROVEAN	SIFT	ACMG guidelines	PM2
Score	1	—	—	PVS1+PM2	—
Prediction	1. Nonsense‐mediated mRNA decay	—	—	Deleterious	Low‐frequency variant
	2. Amino acid sequence change				
	3. Frameshift mutation				
	4. Protein features (likely) affected				

To assess the structural impact of this frameshift, we modeled the mutant ARR3 protein using UCSF Chimera. Structural prediction showed that translation is prematurely terminated at Residue 241 in the mutant, resulting in loss of all amino acids beyond Position 241 and a marked alteration of the C‐terminal protein conformation (Figure 2F–G). Consistent with its functional importance, evolutionary conservation analysis based on Ensembl data demonstrated that the tyrosine at Position 241 is highly conserved across multiple species (Figure 2H). Taken together, these findings suggest that the ARR3 c.721delT frameshift mutation disrupts conserved structural domains and normal protein translation, and is likely a key driver of disease progression in this high myopia family. Schematic representation of the wild‐type and predicted truncated ARR3 proteins, indicating the variant position, the affected downstream C‐terminal region, and the antibody‐recognition epitope located at amino acid Residues 350–388.

### 3.3. Functional Validation of the ARR3 c.721delT (p.Y241Ifs∗3) Mutation in Cultured Cells

**Figure 3:**
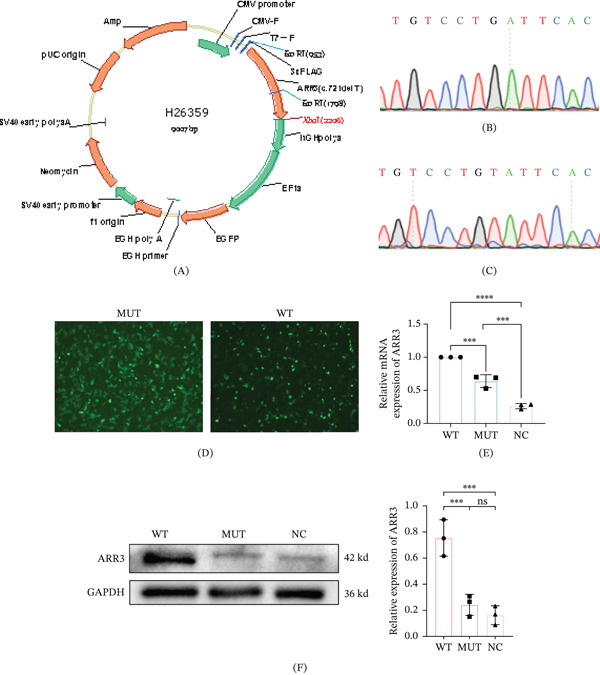
Functional validation of the ARR3 c.721delT (p.Y241Ifs∗3) mutation in cultured cells. (A) Schematic diagram of the ARR3 expression plasmid constructs. (B) Sanger sequencing result of the mutant (MUT) plasmid carrying the ARR3 c.721delT (p.Y241Ifs∗3) variant. (C) Sanger sequencing result of the wild‐type (WT) ARR3 plasmid. (D) Representative fluorescence images of ARPE‐19 cells 24 h after transfection with WT or MUT plasmids, acquired under an inverted fluorescence microscope (40× magnification; scale bar, 200 *μ*m). (E) Quantitative RT–qPCR analysis of ARR3 mRNA expression in ARPE‐19 cells 24 h after transfection. (F) Western blot analysis of ARR3 antibody‐detected signal in ARPE‐19 cells 24 h after transfection and corresponding densitometric quantification. ns, *p* > 0.05;  ^∗^ *p* < 0.05;  ^∗∗^ *p* < 0.01;  ^∗∗∗^ *p* < 0.001;  ^∗∗∗∗^ *p* < 0.0001.

To functionally validate the ARR3 c.721delT (p.Y241Ifs∗3) variant, we constructed expression plasmids carrying wild‐type ARR3 and mutant ARR3 c.721delT. After bacterial culture and endotoxin‐free plasmid preparation, the plasmids were subjected to Sanger sequencing, which confirmed the presence of the intended mutation in the MUT construct (Figure 3A–C).

ARPE‐19 cells were thawed, expanded, and transfected with the indicated plasmids. At 24 h posttransfection, inverted fluorescence microscopy revealed robust green fluorescence in transfected cells. Successful EGFP expression from the transfected constructs was confirmed (Figure 3D). We then assessed ARR3 mRNA expression and ARR3 immunoreactivity. RT–qPCR showed that ARR3 mRNA expression was markedly lower in the MUT group than in the WT group (Figure 3E). Western blotting further revealed decreased ARR3 immunoreactivity in the MUT group (Figure 3F), with highly significant differences between groups (*p* < 0.0001).

These findings provide preliminary expression‐level functional evidence that the ARR3 c.721delT (p.Y241Ifs∗3) variant affects ARR3 transcript abundance and ARR3 immunoreactivity in a heterologous overexpression model. However, direct assessments of cellular injury, cell viability, apoptosis, organelle stress, and autophagy–lysosome activity were not performed in the present study.

## 4. Discussion

In this study, we identified a novel de novo frameshift mutation, ARR3 c.721delT (p.Y241Ifs∗3), in a multigenerational family with eoHM. Pedigree cosegregation analysis, in silico structural prediction, and in vitro functional assays collectively suggest that this variant exerts a severe loss‐of‐function effect and contributes to disease pathogenesis, partially challenging the previously proposed “X‐linked, female‐limited” inheritance pattern of ARR3‐associated high myopia.

eoHM is considered a subtype of refractive error with a particularly strong genetic burden. Epidemiological and pedigree‐based studies consistently indicate marked familial aggregation and stable modes of inheritance in such patients, most commonly autosomal dominant, autosomal recessive, and X‐linked patterns [22–24]. Compared with common myopia, which is more profoundly influenced by environmental factors, eoHM typically presents in early childhood with pronounced axial elongation and structural alterations in the posterior pole, implying a more prominent role of monogenic defects in the regulation of ocular growth and development [25]. Against this background, systematic genetic screening in eoHM families is instrumental in pinpointing novel disease genes and pathogenic loci [26, 27].

Guided by this rationale, we performed whole‐exome sequencing in a family with eoHM and combined the results with cosegregation analysis. Among multiple candidate genes, ARR3 emerged as one of the most plausible causative genes [22, 23], in keeping with the characteristic clinical features and inheritance pattern observed in this pedigree (Figure 1, Table 2). ARR3 mutations have been repeatedly reported in association with X‐linked high myopia, and the cone‐associated arrestin encoded by ARR3 plays a key role in the regulation of cone function, making ARR3 an important candidate gene in eoHM research [28]. In this context, we identified a rare frameshift mutation, c.721delT, in ARR3. This single‐nucleotide deletion introduces a premature stop codon and is predicted to cause marked disruption of the protein structure, thereby providing a foundation for subsequent mechanistic investigations at both structural and functional levels.

In previously reported families with ARR3‐associated high myopia, the mutation spectrum has been dominated by missense variants and nonsense mutations located near the terminal coding region, in which the overall protein structure is at least partially preserved. Clinically, these pedigrees typically show high myopia predominantly in female patients, whereas hemizygous males are often asymptomatic carriers or exhibit only mild refractive changes [29]. In contrast, the c.721delT variant identified in this study is a frameshift mutation located in the midcoding region. A single‐nucleotide deletion is sufficient to alter all downstream amino acids and introduce a premature termination codon (Figure 2D,E), resulting in a truncating variant with a much more profound impact on protein structure. Structural prediction indicates that this mutation leads to loss of the conserved C‐terminal domain of ARR3 and alters local secondary structure and three‐dimensional conformation (Figure 2F,G). In theory, such changes are more likely to cause complete or near‐complete loss of function, which is consistent with the observation that truncating mutations often have more severe pathogenic effects in a range of inherited retinal diseases [30, 31].

With respect to sex distribution, the pedigree described in this study differs markedly from previously reported ARR3 families. Earlier reports have suggested that ARR3‐related high myopia predominantly affects females and may even represent an “X‐linked, female‐limited” form of dominant myopia [32, 33]. In our family, female carriers of the c.721delT frameshift variant exhibited congenital or eoHM, with some patients also presenting with lens opacity or cataract; macular involvement was observed in elderly female patients. In contrast, the hemizygous male was not an asymptomatic carrier but was affected by myopia, although he had a relatively lower refractive error, later onset of high myopia, and no obvious additional ocular complications. These observations suggest that male patients in this pedigree may present with a milder phenotype than female patients, while still expanding the recognized phenotypic spectrum associated with ARR3 variants clinical spectrum of ARR3‐associated myopia. Integrating the mutation type and structural prediction, a plausible explanation is that the frameshift truncation causes such a severe loss of ARR3 function that hemizygous males, lacking a second functional allele for compensation, can also reach the threshold for expressing a dominant phenotype, thereby breaking the previously described pattern in which “males are mostly asymptomatic carriers.”

The difference in sex distribution is unlikely to be fully explained by mutation type alone and may also reflect the combined influence of X‐chromosome inactivation patterns, additional modifier genes, and environmental exposures [34, 35]. On the basis of current data, these remain reasonable hypotheses rather than definitive conclusions, but they at least indicate that the inheritance pattern of ARR3‐related high myopia is more complex than previously appreciated. The frameshift mutation reported in this study provides new clues for understanding this complexity.

To obtain functional evidence beyond sequence and structural predictions, we established an in vitro overexpression model using wild‐type and mutant ARR3 constructs. In this system, the c.721delT mutant construct was associated with reduced ARR3 mRNA expression and a decreased ARR3‐related immunodetected signal compared with the wild‐type construct (Figure 3E,F). These findings suggest that the variant may affect ARR3 expression or protein stability and are consistent with a likely loss‐of‐function mechanism, which is biologically plausible given the established role of visual arrestins in cone phototransduction shutoff and cone function [36]. Because the ARR3 antibody used in this study was not independently validated for recognition of the predicted truncated protein, the Western blot result should be interpreted as indicating a decrease in ARR3‐related immunodetected signal rather than as a direct quantitative measure of truncated ARR3 protein abundance. Moreover, the present experiments were designed primarily to evaluate ARR3 expression after transfection and did not include dedicated assays of cellular injury, cell viability, apoptosis, endoplasmic reticulum stress, autophagy–lysosome activity, or mitochondrial function. Therefore, the current data should not be interpreted as direct evidence of cellular injury or subcellular damage.

In the present in vitro overexpression model, fluorescence microscopy was used primarily to confirm transfection efficiency rather than to provide quantitative evidence of cellular morphology or injury. Accordingly, the microscopic observations were not interpreted as direct evidence of cellular vacuolization, cellular stress, or subcellular damage. Together with the structural prediction and expression data, the in vitro findings support the possibility that the c.721delT frameshift variant affects ARR3 expression and may be consistent with a loss‐of‐function mechanism. However, whether this variant induces cellular stress, organelle dysfunction, or other downstream cellular changes requires further validation using dedicated functional assays, particularly assays targeting endoplasmic reticulum stress and retinal stress‐response pathways [37, 38].

This limitation is important because ARPE‐19 cells constitute a heterologous overexpression system rather than a cone photoreceptor model, and fluorescence microscopy in the present study was used mainly to confirm transfection efficiency. Further studies using cone‐relevant cellular models, retinal organoids, or in vivo systems, together with specific assays of protein degradation, cellular stress, and phototransduction‐related pathways, are required to define the downstream cellular consequences of the ARR3 c.721delT variant [39, 40].

From a clinical perspective, the identification of the ARR3 c.721delT (p.Y241Ifs∗3) frameshift mutation in this study expands the etiological spectrum of eoHM and highlights the need for systematic genetic testing in eoHM families in whom the phenotype cannot be readily explained by traditional environmental factors. In such families, ARR3 and similar genes should be incorporated into routine screening panels [18]. For genetic counseling, our findings indicate that in pedigrees carrying ARR3 variants, attention should be directed not only to the risk in female offspring but also to the possibility of a high‐myopia phenotype in hemizygous males. This has implications for designing sex‐specific follow‐up and intervention strategies. In addition, consideration of skewed X‐chromosome inactivation and its molecular assessment may assist in risk stratification and in explaining the variable expressivity of ARR3 variants [19].

From the standpoint of disease pathogenesis, the association between a predicted truncating ARR3 variant and the high‐myopia phenotype, together with reduced ARR3 mRNA expression and decreased ARR3‐related immunodetected signal in our in vitro model, supports a pathogenic model in which the variant may impair cone arrestin function. However, the downstream cellular events linking ARR3 dysfunction to abnormal ocular growth remain to be clarified. This hypothesis is consistent with the key role of visual arrestins in terminating cone phototransduction and maintaining visual function [41–43]. In the future, the use of single‐cell transcriptomics, multiomics approaches, and human ocular cell atlases may allow more precise mapping of the ARR3‐related network and the cellular compartments involved [44–46]. At the same time, incorporating protein structural prediction and model quality assessment into the evidence framework may enhance structural‐level interpretation of variant pathogenicity [47, 48].

With regard to therapeutic and translational prospects, ocular gene therapy has already been shown to improve functional vision in RPE65‐associated inherited retinal dystrophy, with evidence of durability and acceptable safety, providing a real‐world paradigm for precise gene‐based intervention [49, 50]. Continuous advances in AAV vectors, novel delivery systems, and gene‐editing strategies are laying the technical groundwork for future targeted therapies for monogenic subtypes of myopia [51, 52]. Nevertheless, for the present study, conclusions are mainly derived from a single pedigree and in vitro models, and there remains a considerable gap between these findings and clinically applicable targeted therapies. More systematic data from animal models, natural history studies, and safety evaluations will be required.

In this multigeneration family with eoHM, we identified a novel ARR3 frameshift variant, c.721delT (p.Y241Ifs∗3). Integrated evidence from familial cosegregation, whole‐exome sequencing, in silico pathogenicity assessment, protein structure modeling, and ARR3 overexpression in ARPE‐19 cells supports ARR3 c.721delT (p.Y241Ifs∗3) as an important pathogenic candidate variant. The in vitro findings indicate reduced ARR3 mRNA expression and decreased ARR3‐related immunodetected signal in the mutant construct, but they do not provide direct evidence of cellular injury or subcellular damage. Within the same pedigree, the variant is associated with disease in both heterozygous females and hemizygous males, thereby broadening the mutational and clinical spectrum of ARR3‐related eoHM and providing new clues to its inheritance pattern. These findings support ARR3 c.721delT (p.Y241Ifs∗3) as an important pathogenic candidate variant for eoHM and suggest that ARR3 should be included in routine genetic testing and counseling for families in whom eoHM cannot be adequately explained by environmental factors. Nonetheless, as this study is based on a single family and a heterologous cell model, further validation in larger familial cohorts, cone‐relevant in vivo or organoid models, and multiomics investigations is needed to confirm the pathogenicity of this variant and clarify its molecular pathways, ultimately informing precise diagnosis and potential gene‐targeted interventions in eoHM.

## Author Contributions

**Yadi Li** performed most of the experiments, formal analysis, data curation, and drafted the manuscript. **Fengwu Teng** recruited and followed up participants and carried out ophthalmic examinations and image evaluation. **Qin Zhu** contributed to experimental design, plasmid construction, cell experiments, and statistical analysis. **Shulan Cui and Wenfu Yang** organized and quality‐controlled imaging examinations and assisted with clinical data verification and manuscript review. **Pengcheng Ma** conceived and supervised the study, provided funding and resources, interpreted the data, and critically revised the manuscript.

## Funding

This work was supported by the Yunnan Provincial Science and Technology Department Science and Technology Planning Project (Grant No. 202301AT070484); The Scientific Research Project of the Yunnan Provincial Department of Education (Grant No. 2025J0018); The Special Fund for Training High‐level Health Science and Technology Talents of Yunnan Province (Grant No. H‐2024078); and the Kunming Municipal Health Science and Technology Talent Training Program (Grant No. 2024‐SW(Discipline Leader)‐11); and the Kunming Municipal Health Commission Health Research Project (Grant No. 2025‐02‐02‐001).

## Disclosure

All authors read and approved the final version of the manuscript.

## Conflicts of Interest

The authors declare no conflicts of interest.

## Supporting information


**Supporting Information** Additional supporting information can be found online in the Supporting Information section.

## Data Availability

The data supporting the findings of this study are available from the corresponding author upon reasonable request. Owing to privacy and ethical restrictions related to family‐based clinical and genetic information, individual‐level sequencing data have not been publicly deposited.
