# Supplementary material for: Multilevel Genetic and Functional Assessment of an ARR3 Frameshift Variant in Early‐Onset High Myopia
Source: Int J Genomics. 2026 Jun 20;2026:8894003. doi: 10.1155/ijog/8894003 (PMC13282680; doi:10.1155/ijog/8894003)
Supplement: Supplementary file 1 — Supporting Information Additional supporting information can be found online in the Supporting Information section. [file IJOG-2026-8894003-s001.docx]

Supplementary Table 1 . Representative reported ARR3 variants associated with early-onset high myopia

| Gene | Exon | Patient ID | Nucleotide Change | Amino Acid Change | State | Computed prediction (PPH2/BDGP) | SIF | MAF (gnomAD) | Reported Reference |
| --- | --- | --- | --- | --- | --- | --- | --- | --- | --- |
| ARR3 | 1 | ARR3-001 | c.3G>A | p.Met1? | Hete | D (Damaging) | D | NA | [2] |
| ARR3 | 1 | ARR3-002 | c.7A>T | p.Lys3Ter | Hete | D (Damaging) | D | NA | [11] |
| ARR3 | 1 | ARR3-003 | c.39+1G>A | — | Hete | SSA (splicing site abolished) | D | NA | [10] |
| ARR3 | 1 | ARR3-004 | c.100G>C | p.Asp34His | Hete | D (Damaging) | D | NA | [6] |
| ARR3 | 1 | ARR3-005 | c.100+4A>G | p.Asp34Glyfs*16 | Hete | SSA (splicing site abolished) | D | NA | [10] |
| ARR3 | 1 | ARR3-006 | c.103G>A | p.Gly35Ser | Hete | D (Damaging) | D | NA | [2] |
| ARR3 | 2 | ARR3-007 | c.139C>T | p.Arg47* | Hete | D (Damaging) | D | NA | [9] |
| ARR3 | 2 | ARR3-008 | c.146T>G | p.Leu49Trp | Hete | D (Damaging) | D | NA | [2] |
| ARR3 | 2 | ARR3-009 | c.149T>C | p.Phe50Ser | Hete | D (Damaging) | D | NA | [2] |
| ARR3 | 2 | ARR3-010 | c.214C>T | p.Arg72* | Hete | D (Damaging) | D | NA | [5] |
| ARR3 | 2 | ARR3-011 | c.228T>A | p.Tyr76* | Hete | D (Damaging) | D | NA | [6] |
| ARR3 | 3 | ARR3-012 | c.232C>T | p.Gln78* | Hete | D (Damaging) | D | NA | [2] |
| ARR3 | 3 | ARR3-013 | c.239T>C | p.Leu80Pro | Hete | D (Damaging) | D | NA | [1] |
| ARR3 | 3 | ARR3-014 | c.298C>T | p.Arg100* | Hete | D (Damaging) | D | NA | [1] |
| ARR3 | 4 | ARR3-015 | c.345G>C | p.Gln115His | Hete | D (Damaging) | D | NA | [2] |
| ARR3 | 4 | ARR3-016 | c.346-2A>T | — | Hete | SSA (splicing site abolished) | D | NA | [2] |
| ARR3 | 4 | ARR3-017 | c.361C>A | p.Pro121Thr | Hete | D (Damaging) | D | NA | [2] |
| ARR3 | 5 | ARR3-018 | c.386_389del | p.Asp129Alafs*4 | Hete | D (Damaging) | D | NA | [2] |
| ARR3 | 6 | ARR3-019 | c.484C>T | p.Arg162Trp | Hete | D (Damaging) | D | NA | [7] |
| ARR3 | 6 | ARR3-020 | c.499A>T | p.Lys167* | Hete | D (Damaging) | D | NA | [2] |
| ARR3 | 6 | ARR3-021 | c.520G>T | p.Glu174* | Hete | D (Damaging) | D | NA | [2] |
| ARR3 | 6 | ARR3-022 | c.520delG | p.Val174* | Hete | D (Damaging) | D | NA | [2] |
| ARR3 | 7 | ARR3-023 | c.569C>G | p.Ser190* | Hete | D (Damaging) | D | NA | [8] |
| ARR3 | 9 | ARR3-024 | c.666delC | p.Asn222Lysfs*22 | Hete | D (Damaging) | D | NA | [8] |
| ARR3 | 10 | ARR3-025 | c.707C>G | p.Thr236Arg | Hete | D (Damaging) | D | NA | [2] |
| ARR3 | 10 | ARR3-026 | c.757delC | p.Gln253Argfs*7 | Hete | D (Damaging) | D | NA | [2] |
| ARR3 | 10 | ARR3-027 | c.767+1G>A | — | Hete | SSA (splicing site abolished) | D | NA | [4] |
| ARR3 | 11 | ARR3-028 | c.844_845insT | p.Arg282Leufs*10 | Hete | D (Damaging) | D | NA | [2] |
| ARR3 | 11 | ARR3-029 | c.848delG | p.Gly283Alafs* | Hete | D (Damaging) | D | NA | [4] |
| ARR3 | 11 | ARR3-030 | c.893C>A | p.Ala298Asp | Hete | D (Damaging) | D | NA | [1] |
| ARR3 | 12 | ARR3-031 | c.928G>T | p.Glu310* | Hete | D (Damaging) | D | NA | [2] |
| ARR3 | 12 | ARR3-032 | c.929_930del | p.Glu310Alafs*6 | Hete | D (Damaging) | D | NA | [2] |
| ARR3 | 12 | ARR3-033 | c.963_964del | p.Asn321Lysfs*5 | Hete | D (Damaging) | D | NA | [2] |
| ARR3 | 13 | ARR3-034 | c.989+1G>A | — | Hete | SSA (splicing site abolished) | D | NA | [6] |
| ARR3 | 13 | ARR3-035 | c.1014-2A>G | — | Hete | SSA (splicing site abolished) | D | NA | [3] |
| ARR3 | 14 | ARR3-036 | c.1198C>T | p.Arg400Cys | Hete | D (Damaging) | D | NA | [4] |

1. Xiao X, et al. X-linked heterozygous mutations in ARR3 cause female-limited early onset high myopia. Mol Vis, 2016;22:1257–1266.
2. Wang Y, et al. Genetic and clinical landscape of ARR3-associated MYP26. Br J Ophthalmol, 2022;106:1297–1304.
3. Jiang L, et al. Comprehensive mutation spectrum of ARR3 in Chinese cohorts. Invest Ophthalmol Vis Sci, 2022;63:29
4. van Mazijk AEC, et al. Early onset X-linked female limited high myopia. Ophthalmic Genet, 2022;43:399–405
5. Széll N, et al. Myopia-26 in a European family. Ophthalmic Genet, 2021;42:397–402.
6. Gu L, et al. ARR3 mutation for high myopia and color vision defect. Mol Vis, 2023;29:211–220.
7. Haarman J, et al. Novel ARR3 missense variant in a family with X-linked female-limited high myopia. Ophthalmic Genet, 2022;43:789–793.
8. Xiao X, et al. Novel frameshift variant c.666delC in ARR3. Genes, 2023;14:835.
9. Ye L, et al. Trio-based WES identifies c.139C>T in ARR3. BMJ Open Ophthalmol, 2024;9:e001720.
10. Niu J, et al. Novel splicing variants in ARR3. Mol Vis, 2024;30:112–120.

[11] Gu L, et al. Novel nonsense mutation c.7A>T in ARR3. Eur J Med Genet, 2026;69:104967.
